# Supplementary material for: The causal relationship between the human gut microbiota and pyogenic arthritis: a Mendelian randomization study
Source: Front Cell Infect Microbiol. 2024 Nov 26;14:1452480. doi: 10.3389/fcimb.2024.1452480 (PMC11629706; doi:10.3389/fcimb.2024.1452480)
Supplement: Supplementary file 1 [file DataSheet1.zip › mendelian test/ebi-a-GCST90027617.csv_leave_one_out.pdf]

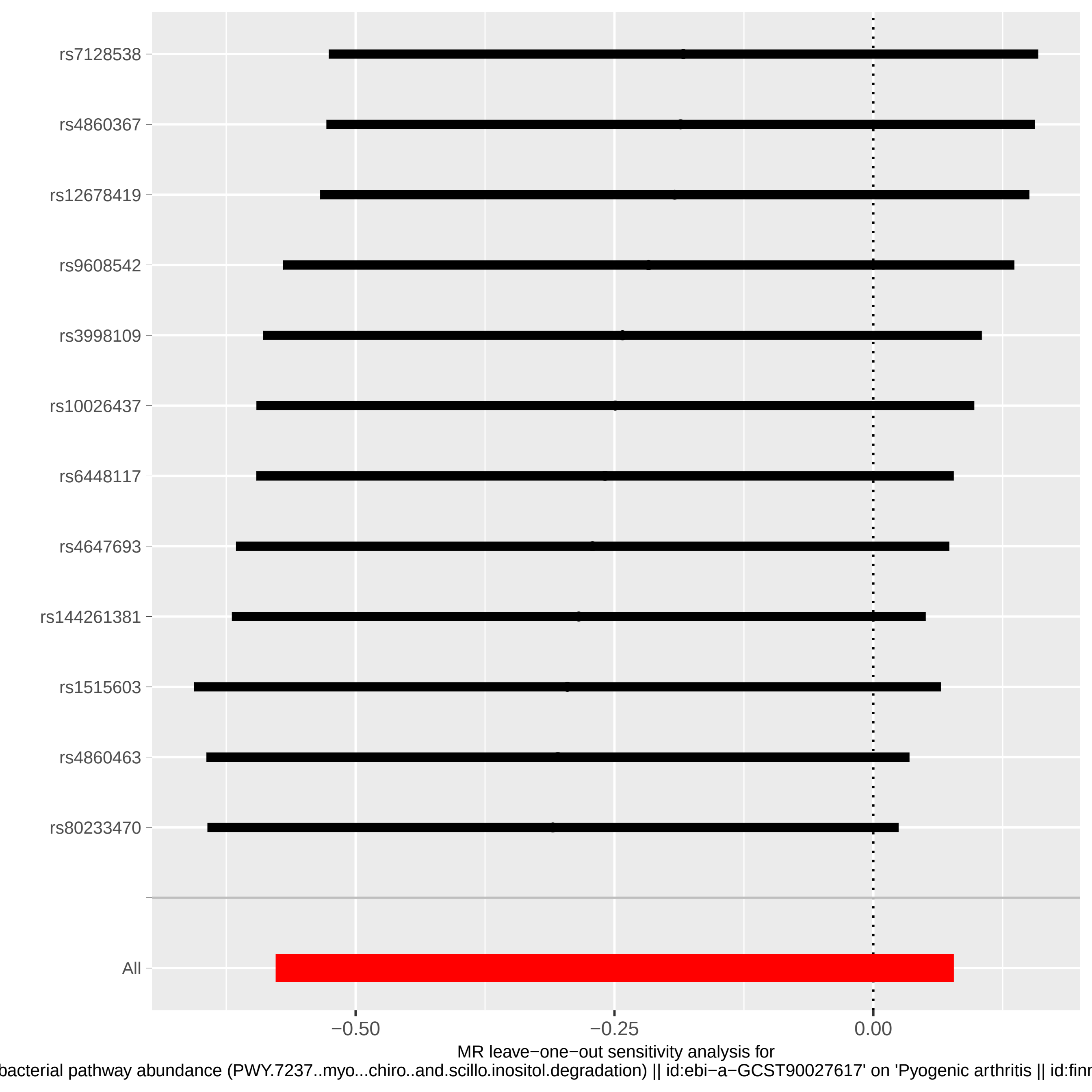
rs7128538
rs4860367
rs12678419
rs9608542
rs3998109
rs10026437
rs6448117
rs4647693
rs144261381
rs1515603
rs4860463
rs80233470
All
−0.50
−0.25
0.00
MR leave−one−out sensitivity analysis for
bacterial pathway abundance (PWY.7237..myo...chiro..and.scillo.inositol.degradation) || id:ebi−a−GCST90027617' on 'Pyogenic arthritis || id:finn
